# Investigating the properties of PLA‐nanochitosan composite films containing *Ziziphora Clinopodioides* essential oil and their impacts on oxidative spoilage of *Oncorhynchus mykiss* fillets

**DOI:** 10.1002/fsn3.2053

**Published:** 2021-01-09

**Authors:** Nastaran Shakour, Zhaleh Khoshkhoo, Afshin Akhondzadeh Basti, Ali Khanjari, Peyman Mahasti Shotorbani

**Affiliations:** ^1^ Department of Food Science and Technology North Tehran Branch Islamic Azad University Tehran Iran; ^2^ Department of Food Hygiene Faculty of Veterinary Medicine University of Tehran Tehran Iran; ^3^ Department of Food Quality Control and Hygiene Science and Research Tehran Branch Islamic Azad University Tehran Iran

**Keywords:** active packaging, mechanical characteristics, polylactic acid, rainbow trout fillets, scanning electron microscopy

## Abstract

The present study aimed to investigate the impact of polylactic acid (PLA) integrated with nanochitosan (NC) composite film and Ziziphora Clinopodioides essential oil (ZCEO) on oxidative spoilage of rainbow trout fillets during a 9‐day refrigeration period. For this purpose, first, six degradable films including T1: PLA, T2: PLA + NC, T3: PLA + NC + 1% ZCEO, T4: PLA + NC + 1.5% ZCEO, T5: PLA + 1% ZCEO, and T6: PLA + 1.5% ZCEO were prepared. Next, the properties of the films were investigated. The results of the mechanical tests showed that ZCEO decreased the tensile strength and increased the elongation at the break of the PLA films. However, adding NC improved the mechanical characteristics of the PLA film. The outcomes of physical tests including moisture content (14.02%), solubility (17.41%), water vapor permeability (1.14 × 10^−7^ g s^−1^ m^−1^ Pa^−1^), and oxygen transmission rate (21.54 cm^3^.mm/m^2^.24 hr) showed that adding 1% ZCEO improved the film physical characteristics. Nevertheless, by adding 1.5% concentration EO to the PLA film, the values of water vapor permeability, porosity (according to the scanning electron microscopy), and turbidity increased and cross‐sectional pores were observed. Moreover, the films had high antioxidant properties (84.33%). In the next step, the effects of the mentioned films on the oxidative spoilage of rainbow trout fillets were investigated. The results of the chemical analysis in PLA film with the EO compared with the control treatment revealed the increasing trend of oxidation indices, in general. Moreover, increasing the concentration yielded better results such that all treatments containing EO showed satisfactory results up until the storage period ends. In most cases, adding NC affected the mentioned characteristics positively, and the best results were observed in T4 and then in T3. However, based on economics and the better mechanical and physical properties of T3, the film can be applied as an active packaging in fishery products.

## Graphical abstract

Rainbow trout is one of the fish widely consumed in Iran and the world. Moreover, fresh fish have a shorter shelf life and are considered as highly vulnerable foods compared to other meat products. Hence, it is essential and beneficial to use proper substances with antioxidant and antibacterial activity to improve the quality and enhance the fish shelf life. The results of this study about the quality of fish fillets indicated that the composite film of PLA‐NC with ZCEO decelerated the increasing trend of oxidative spoilage indices over time while yielding better results by increasing the concentration of ZCEO.

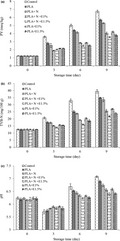

## 1 INTRODUCTION

Fish as an important portion of the human diet is rich in protein, fat, omega‐3, acids, vitamins, selenium, and calcium. Rainbow trout (*Oncorhynchus mykiss*) is one of the fish widely consumed in the world. This fish, which belongs to the Salmonidae family, is a cold‐water fish that is raised in many parts of the world because of its adaptability to different conditions. Because of the high quality and the high nutritional value of this fish, its demand has increased in the world markets over the past decade (Khadem, 2020). However, fresh fish has a short shelf life and is a highly vulnerable food compared to other meat products due to its high muscle pH (pH > 6), high nonprotein nitrogen (NPN) contents, high levels of unsaturated fatty acids, and autolyzing enzymes (Karami et al., 2019; Mohajerfar et al., 2017). Hence, it is essential and beneficial to use proper substances with antioxidant and antibacterial activities to improve the quality, enhance the shelf life, and lower the economic loss associated with this fish. Today, consumers tend to use products of natural origin and with minimal processing. An acceptable and safe approach for prolonging the food shelf life and safety is to utilize plant essential oils (EOs). *Ziziphora* belongs to the *Lamiaceae* family with the general name of *Ziziphora* and an herbaceous species known as *Z. clinopodioides*. This plant grows in Turkmenistan, Armenia, Afghanistan, Anatolia, Syria, Central Asia, Pakistan, the Caucasus, and western Siberia, in addition to Iran. The EO of this plant has been studied by several researchers in terms of its constituents and medicinal effects. The most important antimicrobial compounds in the EO of this plant are terpenoids and phenolic acids, including 1,8‐cineole, thymol, pulegone, and limonene (Mousavian et al., 2020; Shahinfar et al., 2017).

Regardless of the high potential of EOs, their use is limited in maintaining the quality of food, mainly due to their strong aroma and severe toxic problems. One of the interesting options to minimize the required doses is to use biodegradable coatings and films as carriers of these natural compounds. Biodegradable films such as protein and polysaccharide‐based films have recently received more attention for food preservation because of their promising effects (Ekramian et al., 2020; Valipour et al., 2017).

The US Food and Drug Administration have approved polylactic acid (PLA) to be used as packaging material for safe food contacting substance. PLA is derived from the lactic acid monomers of renewable sources, including corn, sugarcane, and acidic whey from fermentation. This acid has some useful characteristics such as high mechanical strength, transparency, and inhibition against the passage of ultraviolet light (Rezaeigolestani et al., 2017). However, this polymer has some disadvantages as well that limit its use as a substitute for synthetic polymers in the food packaging industry. Low thermal stability, high brittleness, and poor inhibitory characteristics against moisture and oxygen transmission are among these disadvantages. Different methods have been proposed to solve these shortcomings. One of these methods is to use nanoparticles and produce nanocomposite polymers (Esfahani et al., 2020; Heydari‐Majd et al., 2019; Shahbazi, 2016).

Chitosan, after cellulose, is the furthermost abundant natural polysaccharide. This compound has many advantages and applications in various industries because of its multiple properties such as biodegradability, biocompatibility, and nontoxicity. One of the significant characteristics of chitosan is its anti‐spoilage and antifungal properties for long storing while maintaining the fresh product and preventing the growth of bacteria (Mahdavi et al., 2018; Qi et al., 2004; Valipour et al., 2017). Nanochitosan (NC) is a natural substance with excellent physicochemical properties that can be used to reinforce the functional properties of polymers, especially to enhance the tensile strength (TS), crystallinity levels, and useful melting temperature of polymers. Using these nanoparticles along with food packaging films is very promising because of the food‐compatible properties (Kazemi et al., 2020; Rezaeigolestani et al., 2017).

Therefore, the current research aimed to examine the physicomechanical properties of PLA film containing NC and *Ziziphora Clinopodioides* EO (ZCEO) and their impact on oxidative spoilage of rainbow trout fillets in the chilled storage period.

## 2 MATERIALS AND METHODS

### 2.1 Raw material

The leaves of *Ziziphora clinopodioides* were collected from Kashan, Iran, in April 2019. They were separated and dried immediately after washing, followed by the scientific label approval by the Institute of Pharmacology. Then, they were dried in a vacuum oven at 50°C for 45 min and completely pulverized by a crusher and kept at 25°C until the experiment.

Noanonovin Polymer Mazandaran Co., provided NC with the deacetylation degree of 80%–85% and molecular weight of 50,000–100,000 Da. PLA granules were prepared from FkuR Kunst Stoff GmbH. Other utilized chemicals, which had a degree of decomposition, were prepared from the Merck Co.

### 2.2 Preparation and analysis of ZCEO

The EO was extracted by a Clevenger‐type device (FarhanTajhiz) as recommended by the European Pharmacopoeia method summarized in the following. Air‐dried and crushed plant leave samples (100 g) with 1,000 ml of distilled water were placed in the Clevenger and hydro‐distillate for almost 3.5 hr. Then, the resultant EO was kept in dark and refrigerated (Shahbazi, 2016).

### 2.3 PLA film preparation

The PLA films were prepared by the casting process based on the method defined by Shahbazi et al. (2016) as described in the following. First, PLA film solution (2%) was prepared by dissolving 2 g of PLA granules in 100 ml of chloroform by striation on IKA magnetic stirrers hot plate (IKA) at room temperature for about 8 hr. Then, the ZCEO (1 and 1.5% v/v) and NC (1% v/v) were added to the solution and homogenized by WISD homogenizer for 1 min at 12,000 g. The final film‐forming solution was then transferred to the glass plates (11 cm diameter), allowing to evaporate the solution overnight at ambient temperature (25°C) (Salmieri et al., 2014).

### 2.4 Measuring film physical properties

#### 2.4.1 Measuring film thickness

A digital micrometer (0.001 mm; Mitutoyo) was used to measure the thickness of samples. In each sample, the measurements were reiterated at five points (Mei et al., 2020).

#### 2.4.2 Measuring film moisture content

The film was cut to 3.3 mm pieces and weighed separately. The measured rate was selected as the primary weight. Then, the pieces were placed in an oven at 90°C until reaching the final dry weight and then weighed (Mohajerfar et al., 2017).

#### 2.4.3 Evaluating the solubility of the films in water

To determine the solubility, the films were cut into 2.2 cm pieces and then placed in a desiccator containing silica gel for 2 days. The films later were weighed using a scale with an accuracy of 0.001 g and placed in a beaker containing 100 ml of distilled water (Zolfi et al., 2014). The solubility percentage was computed as below:

$$\text{Solubility}\;\text{percentage}=\left(\text{initial}\;\text{dry}\;\text{weight}‐\text{final}\;\text{dry}\;\text{weight}\right)/100\;\text{initial}\;\text{dry}\;\text{weight}$$

#### 2.4.4 Water vapor permeability (WVP)

Water vapor permeability of the films was estimated following ASTM Standard E96/E96M‐16 (ASTM, 2016) with some modifications. Water was poured into the glass cells. The thicknesses of the film samples were measured and the cell surfaces were then covered with them and sealed with molten paraffin. Next, the cells were located in a silica gel‐containing desiccator. The weights of the cups were recorded every day for up to 7 days. A digital scale with an accuracy of 0.0001 g was used to measure the changes in cell weight over time. Five samples per treatment were analyzed. Water vapor permeability of the films were obtained by multiplying the film thickness by the steady‐state water vapor transmission and dividing that by film surface area and the water vapor pressure differences of two sides of the cups (Esfahani et al., 2020).

#### 2.4.5 Oxygen transmission rate (OTR)

Permeability to oxygen was measured according to ASTM D 3985. The amount of oxygen transmission was calculated by multiplying the oxygen transfer rate in the uniform flow mode by the average thickness of the film and dividing it by the pressure difference of the two surfaces. The oxygen transfer rate is the amount of oxygen passing through the film's thickness at a given time. The films were conditioned before testing using a desiccator containing saturated magnesium nitrate solution at 25°C (relative humidity of 55%). One of the film surfaces is in contact with the nitrogen atmosphere and the other one contact with oxygen. A barometer sensor mounted on the nitrogen atmosphere side measures the amount of passing oxygen at the nitrogen atmosphere. The experiment is complete when the oxygen concentration on the atmospheric side of the nitrogen reaches a constant level (ASTM, 1996).

ASTM Standard Method D3985‐17 (ASTM, 2017) was applied to evaluate the oxygen transmission rate (OTR) of the films using an OX‐TRAN Model 2/21 10×. First, films were conditioned (RH = 55% and T = 25°C) for 48 hr and then were measured the thickness and mounted into the diffusion cell of the equipment. Transferred oxygen through the films was conducted by the carrier (N2/H2) gas to the colorimetric sensor (Esfahani et al., 2020).

#### 2.4.6 Film opacity

The film samples were cut into rectangles and located inside a spectrophotometer (Thermo Fisher Scientific) cell. The absorption spectrum (200–800 nm) for each sample was recorded by a spectrophotometer, and the film opacity was obtained by the formula below (Peng & Li, 2014):

$$\text{Film}\;\text{opacity}=\text{Absorption}\;\text{at}\;600\;\text{nm}/\text{Film}\;\text{thickness}\;\left(\text{mm}\right)$$

#### 2.4.7 Mechanical properties

Mechanical properties, such as tensile strength (TS) and the percentage of elongation at break of the film samples were evaluated at 25°C with STM‐50 SANTAM (universal tensile and compression testing machine using an ASTM Method D 882). Equilibrated film strips (1 cm × 5 cm) were fixed between the grips with a primary separation of 30 mm, 200 NW load cell, and the cross‐head speed of 5 mm/min. Tensile strength was calculated by dividing the peak load by the cross‐sectional area of the initial film sample. Elongation was obtained by percentile of a change in the length of the sample to the original distance between the grips (30 mm) (Almasi et al., 2014).

#### 2.4.8 X‐ray diffraction (XRD)

X‐ray diffraction is a useful method for examining the crystallographic structure of the film specimen nanomaterials at room temperature. XRD patterns were recorded with a Rigaku X‐ray diffractometer, operating at 40 kV and 40 mA, prepared with Cu Ka radiation at a wavelength of 0.15406 nm. The basal spacing of the silicate layer (d001) was obtained by the Bragg's equation *λ* = 2*d* sin *θ*, where *λ* represents the wavelength of the X‐ray radiation applied (0.1546 nm), *d* shows the spacing between diffraction lattice planes, and *θ* is the measured diffraction angle (Rhim et al., 2009).

#### 2.4.9 Scanning electron microscopy (SEM)

The microstructures of dried films were investigated by preparing their thin sections and monitoring them using an SEM device (with 100‐µM resolution and X500 magnification ability and the voltage of 15 kV). After gold coating, the images were captured using the DSR1 BAL‐TEC SCD 005 sputter coater (BAL‐TEC AG) and FESEM (TESCAN MIRA2 LMU) with an accelerating voltage of 15 kV (Almasi et al., 2014).

### 2.5 Measuring the films' antioxidant activity

The percentage of radical scavenging (2‐2‐diphenyl‐1‐picryl hydroxyl) of DPPH was determined using a standard method with some modifications. About 25 mg of the film was gently stirred in 3 ml of distilled water for 5 min. Then, 8.2 ml of the film extract was added to the test tubes containing 0.2 ml of a 1 mM DPPH solution in methanol and stored for 30 min at room temperature. The absorbance of test and control tubes at 517 nm was measured by a spectrophotometer (Siripatrawan & Harte, 2010). The decolonization degree of this compound indicates the ability to trap free radicals by the relevant antioxidant. Ultimately, the percentage of DPPH free radical scavenging activity was estimated as follows:

$$\text{Percentage}\;\text{of}\;\text{inhibition}\;=\;\frac{\text{control}\;\text{absorption}‐\text{sample}\;\text{absorption}}{\text{control}\;\text{absorption}}\times 100$$

### 2.6 Preparation of treatments

The required 30 rainbow trout fillets with an average weight of 400 ± 50 g were prepared from fish farms in Tehran and transferred to the laboratory under the correct conditions. After cutting, emptying the viscera, skinning, and removing the bones of the fish, the fishes were washed with cold water. Next, about 60 fillets of 100 2 g were prepared and kept in the refrigerator until the experiment. The fillets were then wrapped up in the PLA films containing different concentrations of ZCEO and NC (uncovered fillets were applied as the control) and kept for 9 days at the refrigerated conditions. Each specimen was tested in triplicate.

### 2.7 Chemical analysis

#### 2.7.1 Peroxide value

The number of initial oxidation products (hydroperoxides) is measured by the peroxide value (PV). The method proposed by Pearson was used for determining the peroxide value of specimens (Bagheri et al., 2016). Results were presented in mEq oxygen kg^−1^ lipids.

#### 2.7.2 PH

The pH was obtained by homogenizing 5 g of the fish specimen in 45cc of distilled water. A digital pH meter was then used to measure the pH (Valipour et al., 2017).

#### 2.7.3 Total volatile basic nitrogen (TVB‐N)

The microdiffusion method was used to determine the TVB‐N of the beef. The results were presented as mg N/100 g of specimens (Javadian et al., 2017).

### 2.8 Statistical analysis

Experiments were all carried out in a fully randomized experimental design with three replications, and the results were presented as mean with standard deviation. Statistical analysis of treatments was performed using analysis of variance (ANOVA) IBM SPSS Statistics 22.0 (IBM SPSS, Inc.). The mean significant differences were determined based on Duncan's test at the level of 0.05 and the figures were drawn with Microsoft Excel software.

## 3 RESULTS AND DISCUSSION

### 3.1 Determining the constituents of ZCEO

According to the results related to chemical compounds (Table 1), 45 compounds including 96.48% of EO compounds were identified. Among the constituent compounds, the highest content of the EO compounds was related to thymol (45.52%), acetate (11.61%), and benzene (5.19%). Based on the results, the main ingredient in ZCEO is thymol. These results are in line with those of (Aghajani et al., 2008) who also specified that the main constituent of *Ziziphora clinopodioides* collected from Lorestan (Iran) is thymol (53.6%) and then carvacrol (8.7%). Moreover, it was stated that the main constituents of ZCEO mustered from West Gilan are carvacrol and then thymol (Shahbazi et al., 2016). However, contrary to the above reports, some researchers reported Pulegone as the main ingredient of ZCEO collected from the mountainous areas of Bojnourd (Jafari et al., 2018; Shahinfar et al., 2017). Generally, various studies reported differences in the amount and type of EO compounds. The ingredients of plant EOs can be different based on the geographical area of growth, seasonal and environmental circumstances, plant harvest time, extraction and drying techniques, EO extraction from different organs, and ultimately plant genetic differences (Mahdavi et al., 2018; Shahbazi et al., 2016).

**TABLE 1:** Chemical compounds of Ziziphora essential oil

Row	Components	%	RT
1	Bicyclo[3.1.0]hex‐2‐ene	0.35	6.903
2	1S‐alpha‐Pinene	0.76	7.087
3	Camphene	0.69	7.49
4	beta‐Pinene	0.23	8.279
5	beta‐Myrcene	0.63	8.7
6	Bicyclo[4.1.0]hept‐2‐ene	0.89	9.447
7	benzene	5.19	9.719
8	limonene	0.26	9.82
9	eucalyptol	2.46	9.897
10	1,4‐Cyclohexadiene	3.56	10.727
11	Terpineol	0.25	10.929
12	Cyclohexene	0.23	11.563
13	1,6‐Octadien	3.35	11.961
14	Bicyclo[2.2.1]heptan‐2‐one	0.75	13.223
15	Borneol	2.72	13.887
16	3‐Cyclohexen‐1‐ol	0.99	14.196
17	p‐menth‐1‐en‐8‐ol	1.22	14.581
18	2,6‐Octadien‐1‐ol	0.38	15.619
19	Benzene	0.66	15.802
20	2,6‐Octadienal	0.27	15.986
21	Benzene	1.77	16.075
22	2,6‐Octadien‐1‐ol	2.5	16.395
23	2,6‐Octadienal	0.85	16.988
24	Acetic acid	0.23	17.267
25	Thymol	42.52	17.747
26	Eugenol	0.21	19.223
27	acetate	11.61	20.012
28	Caryophyllene	2.44	20.872
29	1H‐Cycloprop[e]azulene	0.2	21.328
30	Naphthalene	0.22	22.247
31	enzene	0.92	22.384
32	Bicyclogermacrene	0.76	22.757
33	Cyclohexene	0.64	23
34	Naphthalene	0.49	23.172
35	Naphthalene	0.55	23.38
36	Bicyclo[3.1.1]hept‐2‐ene	0.96	23.812
37	Butanoic	0.36	24.186
38	imethyl	0.33	24.287
39	1H‐Cycloprop[e]azulen‐7‐ol	0.5	24.702
40	Caryophyllene	0.76	24.844
41	Butanoic acid	0.41	25.17
42	alpha‐Cubebene	0.14	25.544
43	Naphthalene	0.97	26.131
44	Alpha‐ cadinol	0.15	26.558
45	2,6‐Octadien‐1‐ol	0.15	28.502
			Total: 96.48

### 3.2 Investigating the physical properties of the films

Sensitivity to moisture is a critical property for a biodegradable film. Efforts to lower this sensitivity are valuable for the future application of such materials in packaging industries (Esfahani et al., 2020). Based on the results (Table 2), the highest moisture content was in the PLA film (17.14%). The moisture content decreased with the addition of NC since chitosan absorbs less water than PLA. Therefore, due to its presence in combination with PLA, its hydrophilicity is reduced and the amount of moisture absorption is decreased. Moreover, the placement of NC between the PLA filaments increases the structural cohesion and prevents the film from absorbing high moisture by reducing the free OH groups and reducing the space between the PLA filaments. Furthermore, adding ZCEO reduced the moisture content of the films. This phenomenon can be because of the interchain covalent bonds between PLA and NC and EOs. The formation of these bonds reduces the hydroxyl and free amine groups in the film network and hence reduces the number of hydrogen bonds between the functional groups of the polymer chains and the water molecules. Thus, reducing the hydrogen bonds led to a reduction in the moisture content of films containing ZCEO (Mohajerfar et al., 2017). The lowest amount of moisture was observed in the film containing PLA + NC + 1.5% ZCEO (13.45%). The current research results are consistent with those of Mahdavi et al. (2018) regarding the moisture content in connection with the addition of anise EO to the chitosan film.

**TABLE 2:** Evaluation of physical characteristics of edible films

Film type	Moisture (%)	Solubility (%)	Thickness (mm)	WVP (×10^−7^ g s^−1^ m^−1^ Pa^−1^)	OTR (cm^3^.mm/m^2^.24 hr)	Opacity
PLA	17.14 ± 0.19^a^	20.40 ± 0.50^a^	0.038 ± 0.0005^d^	1.50 ± 0.03^a^	33.58 ± 1.54^a^	0.9 ± 0.02^f^
PLA + N	15.91 ± 0.14^b^	18.19 ± 0.19^b^	0.04 ± 0.001^c^	1.28 ± 0.03^c^	26.98 ± 1.05^b^	0.97 ± 0.02^e^
PLA + N + E1%	14.02 ± 0. 07^cd^	17.41 ± 0.51^c^	0.049 ± 0.001^b^	1.14 ± 0.05^d^	21.54 ± 1.45^d^	1.12 ± 0.03^c^
PLA + N + E1.5%	13.45 ± 0.38^d^	15.47 ± 0.51^e^	0.056 ± 0.002^a^	1.36 ± 0.03^b^	22.45 ± 0.87^d^	1.30 ± 0.03^a^
PLA + E1%	15.53 ± 0.33^b^	16.92 ± 0.11^c^	0.042 ± 0.001^c^	1.27 ± 0.03^c^	24.98 ± 0.75^c^	1.2 ± 0.04^d^
PLA + E1.5%	14.21 ± 0.59^c^	16.15 ± 0.14^d^	0.057 ± 0.001^a^	1.38 ± 0.03^b^	24.55 ± 0.68^c^	1.18 ± 0.03^b^

Note

Significant differences in a same column are shown by different letters (*p* < .05). Values are means ± standard deviation (*n* = 3).

Solubility and moisture content are two important factors of biodegradable films affecting the film's resistance to water, especially in humid environments. The solubility of the film depends on water diffusion, amino and carboxylic groups, separation of hydrogen, and ionic bonds (Peng & Li, 2014). Solubility in water could be regarded as an important factor for estimating the biodegradability of films (Ekramian et al., 2020). The results of the present work regarding solubility (Table 2) and moisture were consistent. The addition of NC to the PLA film reduced the solubility values. The observed reduction in water solubility of the films was due to the formation of strong hydrogen bonds between the hydroxyl biopolymer and NC groups. These bonds enhance the biopolymer matrix cohesiveness, thus reducing the sensitivity of the films to water and thus preventing water molecules to break these strong bonds. In PLA + NC films, the solubility was reduced such that the lowest solubility appeared in the PLA + NC + 1.5% ZCEO (15.47%) films. The hydrophobic properties of EOs caused by their lipid nature resulted in the hydrophobicity of specimens containing EOs and producing a film with less water solubility. Such a film may be beneficial in covering foods with a wet surface, including fish (Bahram et al., 2014).

One of the essential factors of the film is thickness, which directly affects the biological properties and the packaged product durability. The film thickness was used to define WVP and mechanical properties (Mei et al., 2020). Based on the results of the present study related to the thickness (Table 2), the addition of NC and also ZCEO to the PLA film increased the film thickness so that the highest thickness was found in the PLA film + NC + 1.5% ZCEO (0.056 mm) and PLA + 1.5% ZCEO (0.057 mm). The increased thickness can be attributed to the trapping of microdrops of EO in the PLA films; moreover, various chemical compounds in the EO cause the film structure to stick out and increase the film thickness (Dashipour et al., 2014). It has also been stated that adding cellulose nanoparticles to PLA film increases the film thickness (Shahbazi, 2016). Furthermore, adding clove EO to chitosan film increases the thickness of chitosan film (Shahbazi, 2016). Therefore, it is essential to assess the WVP of polymer films when using antimicrobial films in food packaging materials with medium and high humidity. The exchange of gases, flavor, and moisture between food and the environment is one of the reasons for decreasing the food quality (Wihodo & Moraru, 2013). According to the results, the amount of WVP was reduced by adding NC to the PLA film media (Table 2). The presence of NC particles in the biopolymer matrix forms zigzag and tortuous pathways for water vapor molecules to penetrate. These hydrophobic filaments make water vapor molecules travel a longer and more complex path within the film, thereby reducing the rates of transmission and penetration of water vapor molecules (Gao et al., 2011). By adding ZCEO with a concentration of 1% to the PLA film media, the WVP was reduced so that the lowest amount of WVP was observed in the PLA film + NC + 1% ZCEO (1.14 × 10^−7^ g s^−1^ m^−1^ Pa^−1^). EO‐containing films reduce the hydrophilicity of the film and show good performance in preventing water vapor because of their increased hydrophobicity in the films. The WVP of the studied films increased with an increase in the concentration of EO to 1.5%. The reason is that the placement and composition of the EO cause changes in the structure of molecules existing on the polymer surface and reduce the compactness of the surface structure, thereby accelerating the passage of water vapor through the films. Furthermore, using high concentrations of EO may cause discontinuity or so‐called fuzzy separation in the produced films. Such surface changes are confirmed by the SEM mages from the surface of the films since the PLA film without EO showed a smooth and uniform surface. However, the addition of EO decreased the amount of compaction between the films and the formation of pores (Jouki et al., 2014).

In the current research, NC and ZCEO were added to enhance the permeability properties of water vapor and oxygen in PLA films. Improving these properties in PLA will increase the applications of this biodegradable polymer in the food packaging industry. The reason is that one of the most significant factors in the destruction and spoilage of food is the presence of oxygen and water vapor in the interior of their packaging structure. To enhance these properties, compounds with high water absorption power were added to the PLA film to prevent the passage of water vapor. Furthermore, adding NC reduced oxygen transmission rate (OTR) (Table 2). The oxygen permeability depends on factors such as polymer chain flexibility, polymer's phase, physical state, and molecular arrangement. Substrates with flexible polymer chains and high freedom of movement have poor oxygen‐inhibiting properties. NC plays an effective role in reducing oxygen permeability in the PLA substrate, possibly due to the limited range of polymer chain motion to form a rigid three‐dimensional network of NC within the substrate (Silvério et al., 2013). Adding ZCEO to the PLA film, the amount of OTR was reduced so that the lowest amount of OTR was observed in the PLA film + NC + 1% and 1.5% ZCEO (22.45 and 21.54 cm^3^.mm/m^2^.24 hr, respectively). By filling the pores created between the films in the large network with a linear structure and due to the presence of softener in the film composition, the amount of these empty spaces was increased such that a structure with less empty spaces between the networks against gas passage was created. Despite limited information available in this regard, adding EOs reduces the permeability to gases, in general (Choi et al., 2017).

Measuring the visual characteristics is essential to determine the application of films on the food surface because of their direct relation to the appearance of packaged food. Based on the results of the present research, the addition of NC and ZCEO to the PLA film increased the film opacity such that the highest opacity was found in the PLA + NC + 1.5% ZCEO. These changes are probably due to the dark color of the EOs as well as the presence of polyphenolic compounds in the EOs. Furthermore, it can be associated with the increased roughness and thickness of films containing NC and EO, along with the effect of light scattering in contact with the EO. Since thickness is one of the factors affecting the transparency of films, the opacity significantly increases (Łopusiewicz, 2018). The alterations in film transparency may reduce consumer demand. Nevertheless, the reduced film transparency may decrease the transmission of visible and ultraviolet light through the film, resulting in changes in color and taste, loss of nutrients, and ultimately decelerating the oxidative spoilage of food (Arrieta et al., 2014).

### 3.3 Investigating film mechanical properties

Tensile strength (TS) is measured by the maximum stress needed to break the film through the tensile test. In this study, the results related to the TS and tear strength of films (Table 3) were consistent so that the addition of NC to PLA film increased the TS. This result can be attributed to two factors: (a) uniform and optimal bonding and dispersion of polymer nanoparticles and (b) effective stress transfer from polymer to NC surfaces. Indeed, the improvement in the mechanical strength of nanocomposite films can be ascribed to the effective transfer of pressure to the NC reinforcement network, minimization of stress concentration area, and uniform stress distribution (Varanda et al., 2007). NC itself also has a higher mechanical strength compared to PLA and can help strengthen the mechanical properties of the film in this regard. Other studies reported similar conclusions regarding the chitin nanofiber effects on the mechanical properties of starch films (Chang et al., 2010). In the current work, TS was reduced by adding ZCEO to the PLA film samples. Thus, lower TS and tear resistance were observed in the PLA films containing the higher concentration of ZCEO (1.5%). Because EOs act as plasticizers and enhance the flexibility of polymer chains, their presence in films can lead to poor network structure. Therefore, TS may decline while increasing the elongation percentage. These results are in line with those of (Llana‐Ruiz‐Cabello et al., 2016) regarding the addition of oregano EO to PLA film and the results of (Gonçalves et al., 2020) about adding clove EO to chitosan film. The results of maximum tension before the breaking point (Table 3) were inversely related to the results of TS so that by adding ZCEO to the PLA film, the maximum tension increased before the breaking point and the least values of maximum tension before the rupture point were found in the PLA + NC film samples. As mentioned earlier, the presence of EO in the polymer substrate can enhance the mobility and flexibility of the polymer chains by reducing the intermolecular forces in the polymer chains. Therefore, the ZCEO as a plasticizer can increase the length of the film to the breaking point and reduce the tensile strength of the film samples.

**TABLE 3:** Evaluation of mechanical characteristics of edible films

Film type	Tensile strength (MPa)	Tear resistance (MN)	Elongation break (%)
PLA	39.08 ± 1.37^b^	4.26 ± 0.10^a^	2.75 ± 0.05^d^
PLA + N	45.39 ± 0.73^a^	4.86 ± 0.11^b^	2.58 ± 0.04^e^
PLA + N + E1%	37.48 ± 0. 86^b^	4.19 ± 0.02^bc^	2.90 ± 0.04^c^
PLA + N + E1.5%	28.02 ± 1.12^d^	4.03 ± 0.05^c^	2.99 ± 0.03^bc^
PLA + E1%	33.86 ± 0.76^c^	3.96 ± 0.04^c^	3.05 ± 0.05^ab^
PLA + E1.5%	24.75 ± 1.07^e^	3.76 ± 0.06^d^	3.14 ± 0.08^a^

Note

Significant differences in a same column are shown by different letters (*p* < .05). Values are means ± standard deviation (*n* = 3).

### 3.4 Scanning electron microscopy (SEM) test

**FIGURE 1:**
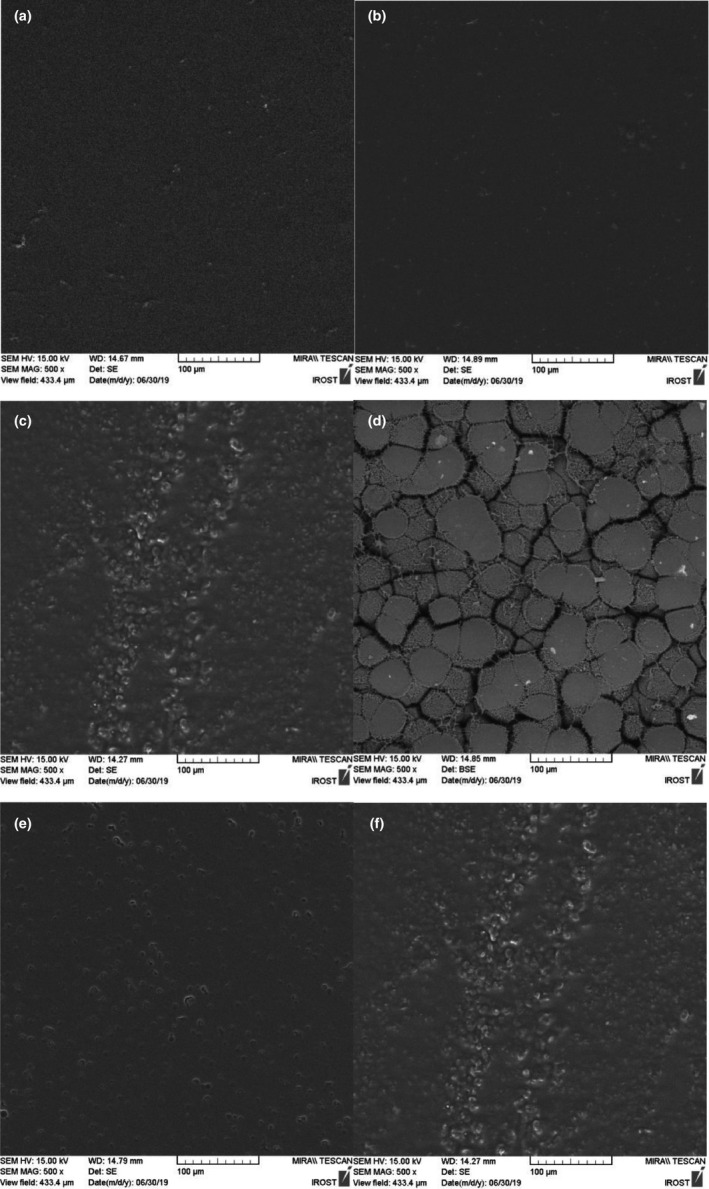
Scanning electron microscopy (SEM) of different types of film [PLA (a), PLA + N (b), PLA + N + E 1% (c), PLA + N + E 1.5% (d), PLA + E 1% (e), PLA + N + E 1.5% (f)]

Investigating the microstructural properties in biopolymers is a vital factor in understanding the behavior and properties of biopolymers. The results of the SEM images in (Figure 1) indicate that the pure PLA film is perfectly smooth and uniform without any bubbles, pores, or cracks. Furthermore, the results of SEM images proved the uniform and appropriate distribution of chitosan nanoparticles in the PLA substrate. The surface of the PLA film reveals that the addition of ZCEO causes heterogeneity and unevenness, which is probably caused by the arrangement of EO molecules during the formation of the film. This can be done in different ways leading to the increased thickness of the films and heterogeneity at the film surface. However, the PLA + NC film containing 1.5% of ZCEO has a sponge‐like structure with holes observable all over the cross‐section of the film. The presence of cavities and porosity in the cross‐section of the film is related to the volatility of the ZCEO, which results in changes in the polymer chain, spherical polymer particles, and space between the matrixes. It ultimately leads to increased roughness, reduced tensile strength, light transmission, and increased WVP in the produced films (Jouki et al., 2014).

### 3.5 X‐ray diffraction (XRD)

**FIGURE 2:**
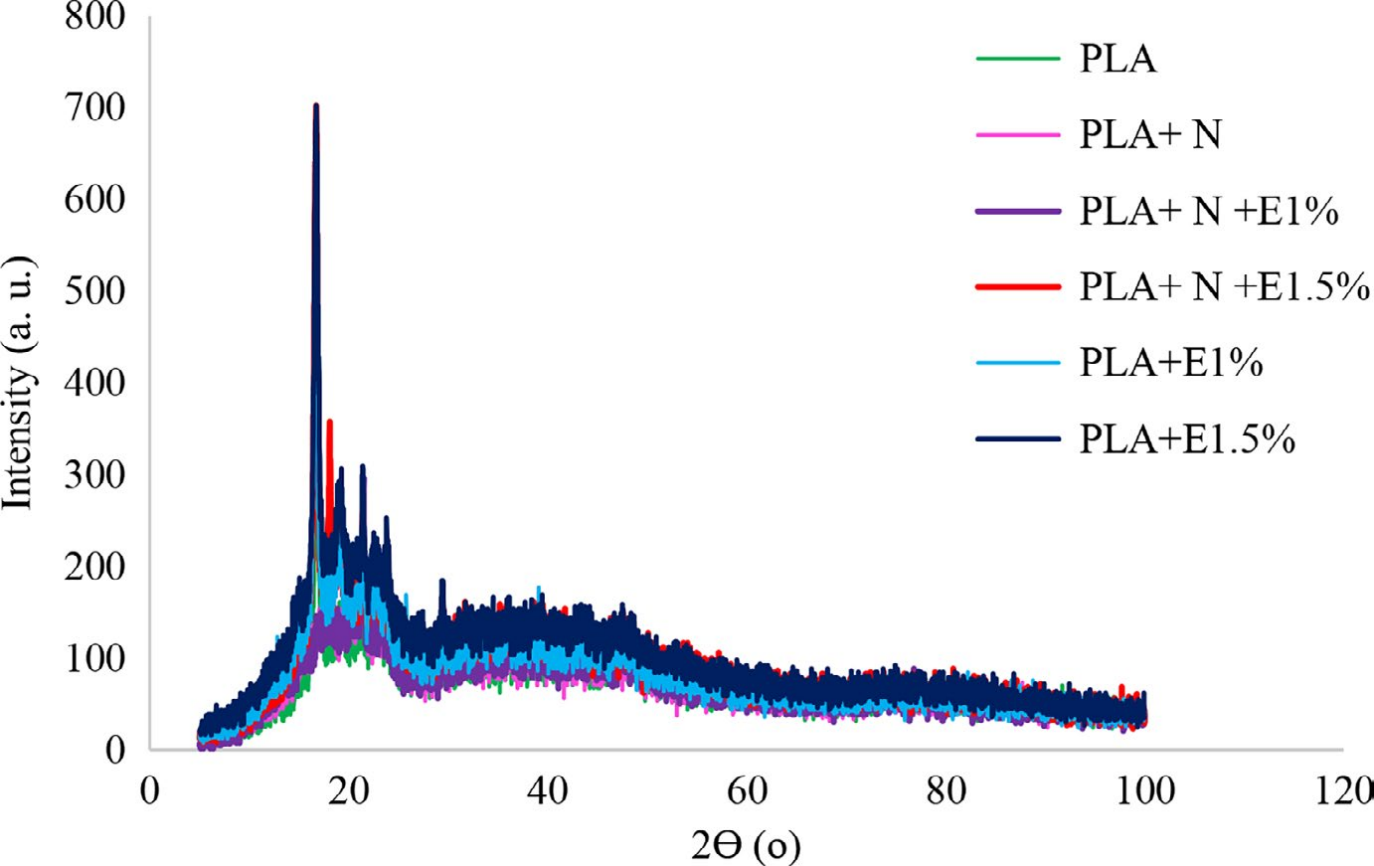
X‐ray diffraction (XRD) of different types of film

According to the XRD test (Figure 2) conducted on different films, all films revealed their longest peak at an angle of 2Ɵ = 18°. In fact, excluding the narrow peak observed at 2Ɵ = 18°, the pure PLA film showed an amorphous nature. Therefore, PLA can be considered as a semi‐crystalline polymer. As shown in Figure 2, there was no significant difference between the diffraction patterns of pure PLA film and NC containing PLA films. So, it can be concluded that NC does not affect the crystalline structure of PLA. This may be due to its low molecular weight and its capability to locate between amorphous areas of the polymer. In films containing ZCEO, the intensity of the peaks increased to 2Ɵ = 18°, suggesting an increase in the crystallinity of the films. Accordingly, it can be concluded that PLA films containing ZCEO have a higher degree of crystallinity as compared with pure PLA films because of the EO effects on increasing density and bonds. Moreover, as expected, with an increase in the EO concentration, the intensity of the peaks increased, indicating an increase in the hydrophobicity of the films and the interaction of the polymer substrate with the ZCEO. Researchers investigated the impact of chitosan‐nanocellulose composite films with different EOs (Zhang et al., 2018). According to the XRD results, they indicated that the crystallization of nanocellulose films could be increased in combination with chitosan‐EO through hydrogen bonds and their electrostatic interactions. Hence, it can be concluded that adding EOs and chitosan to the nanocellulose film increases the intensity of the peaks in the pure film.

### 3.6 Investigating film antioxidant properties

**FIGURE 3:**
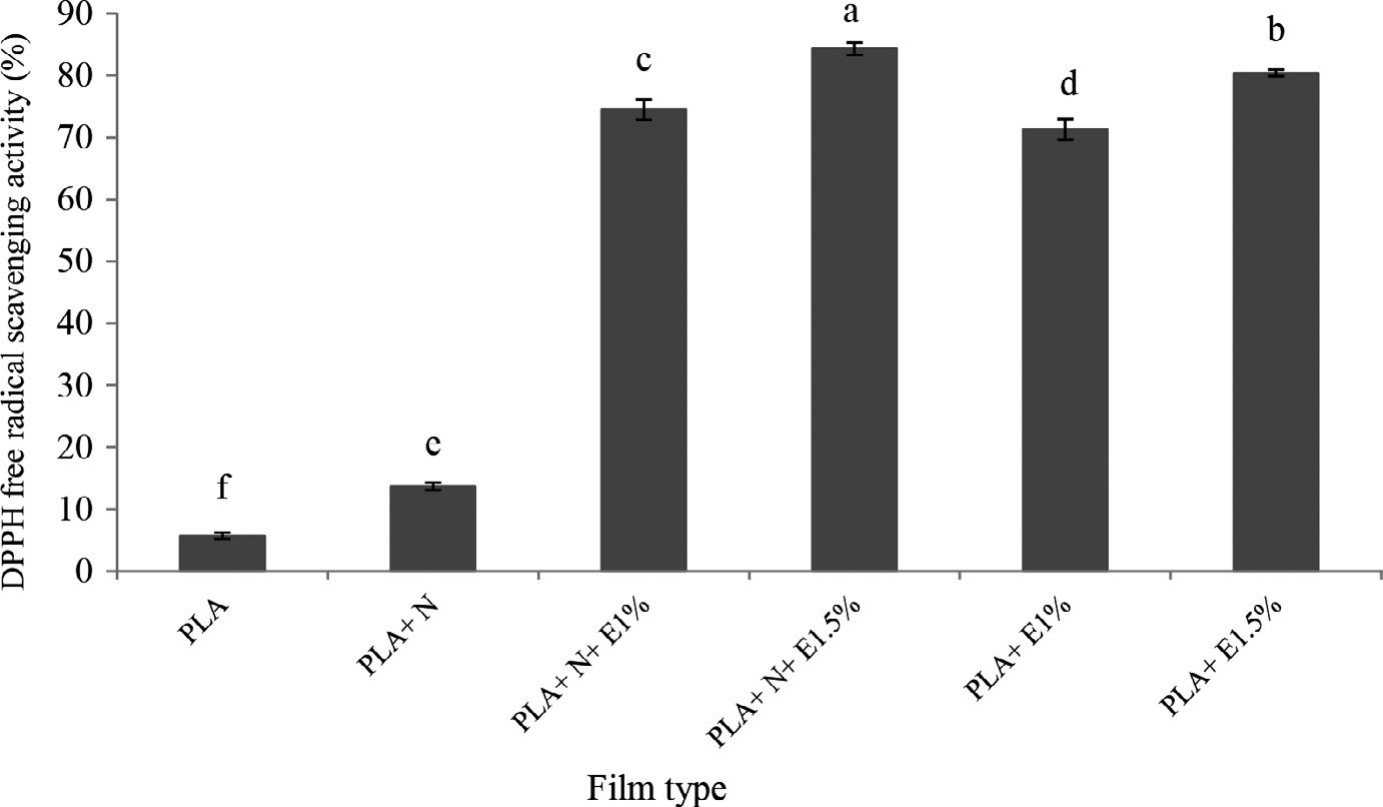
Antioxidant activity (DPPH free radical scavenging activity) of different types of film

Based on the findings, the lowest values of antioxidant properties (Figure 3) were in the pure PLA film samples. By adding NC to the PLA film, the number of antioxidant properties was incremented. In general, chitosan decelerates oxidation reactions by trapping metal ions and prevents the contact between oxygen and the content of the by trapping free radicals if used in food packaging materials (Pothiyappan et al., 2013).

By the addition of ZCEO, the inhibition of the free radical activity of DPPH is also increased. Plant EOs possess antioxidant activity because of their phenolic compounds. The antioxidant activity of phenolic compounds is mostly owing to their oxidation–reduction properties; hence, they act as decreasing agents, oxygen scavengers, and hydrogen donors (Mohdaly et al., 2011). EO radical scavenging activity increases with an increase in the concentration of phenolic compounds. Some other researchers stated that by increasing the concentration of phenolic compounds, the antioxidant property is increased as well (Burt, 2004; Rashidaie Abandansarie et al., 2019).

### 3.7 Investigation of chemical changes of fish fillets during storage

**FIGURE 4:**
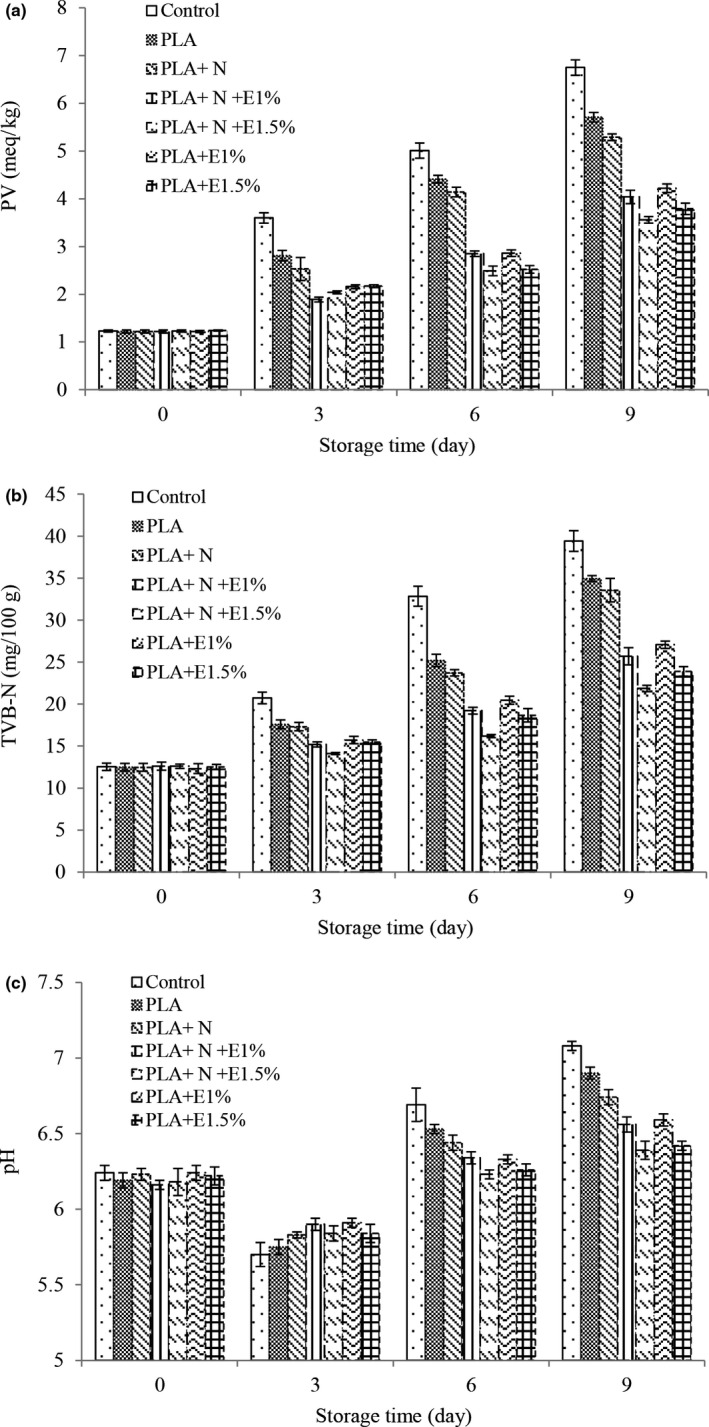
Changes in peroxide value (a), total volatile base nitrogen (b), and pH (c) of different

Fat oxidation is one of the major reasons for spoilage during storage leading to changes in the odor, unpleasant taste, and reduced nutritional value. Peroxide number is used to define the formation of hydroperoxides. Hence, determining the peroxide numbers in fish meat samples seems necessary to determine fat oxidation (Valipour et al., 2017). The results indicated that the peroxide number (Figure 4a) is increased over time in all treatments. Comparing the peroxide numbers of the control sample and other treatments in different storage periods revealed that the treatments containing ZCEO and NC decelerated the increasing trend of peroxide number. The addition of NC to the PLA film slowed the downward trend in the peroxide numbers. Previous studies have shown that Chitosan has an antioxidant activity to retain fats in food. Better results were found by adding ZCEO. The lower peroxide number in these treatments is because of EO high antioxidant properties (Bingöl et al., 2015). ZCEO antioxidant activity is associated with phenolic compounds such as thymol. Thymol effectively neutralizes free radicals such as proxy radicals, superoxide radicals, hydrogen peroxide, and nitric oxide. Thymol exhibits antioxidant activity in both in vivo and in vitro conditions, because of the presence of a hydroxyl agent attached to its aromatic rings. Moreover, the weakly acidic properties of thymol facilitate the reactions of the free radicals (Fizur et al., 2017). By increasing the percentage of EO, its antioxidant properties also increased. The permissible amount of peroxide in fish meat is 5 mEq/kg fat for human consumption (Yanar, 2007). Once the storage period ends, the amount of peroxide had an acceptable range in all treatments containing EO.

In the current study, by increasing the time, pH values (Figure 4b) decreased and then increased in all treatments at the start of the maintenance period. The primary decrease in pH may be caused by the activity of *lactic acid bacteria* and the acidification of the environment. The increased pH throughout the storage period can also be due to the increased formation of volatile nitrogen bases (such as ammonia and trimethylamine) due to the activity of spoilage bacteria (Kim et al., 2011; Rashidaie Abandansarie et al., 2019). Based on the results of statistical analysis, in most of the days, the highest values were detected in the control treatment. Adding NC to the PLA film during most of the storage time decelerated the increase in the pH process. Also, better results were observed by the addition of ZCEO. Furthermore, by increasing the concentration, a positive effect was found in this regard such that on day 9 of storage, the lowest pH values were observed in the treatments of PLA + NC + 1.5% ZCEO and PLA + 1.5% ZCEO. This may be caused by the inhibitory effects of ZCEO on bacterial growth, glycogenolysis, and dissolution of carbon dioxide and its conversion to carbonic acid during storage (Shahinfar et al., 2017).

Measurement of volatile nitrogen bases is a quantitative factor in determining the quantity of ammonia and amines of the first, second, and third types in fish meat. In general, in all treatments, the number of volatile nitrogen bases increased by increasing the time (Figure 4c). The increased volatile nitrogen bases in fish can be due to different enzymatic processes such as deamination of the free amino acids, nucleotide breakdown, and amine oxidation (Valipour et al., 2017). The highest values of volatile nitrogen bases were detected in the control treatment. Adding NC to the PLA film did not affect the increasing trend of volatile nitrogen bases significantly. Nevertheless, the addition of ZCEO decelerated the increasing trend of volatile nitrogen bases and had a positive effect by increasing the concentration. The lower amount of volatile nitrogen bases in this treatment compared to other treatments can be attributed to the reduced bacterial population of these treatments or reduced oxidative capacity of bacteria to separate amines from nonvolatile nitrogen compounds or both, which are caused by the impact of EOs on bacteria in fillets. By increasing the concentration of ZCEO, its antibacterial effect also increased because of the increase in the content of phenolic compounds. Therefore, in the treatment containing the higher concentrations of ZCEO, the number of nitrogen bases was lower (Kim et al., 2011; Rashidaie Abandansarie et al., 2019). Other researchers stated that edible films along with plant EOs have excellent resistance to increasing TVB‐N levels (Echeverría et al., 2018; Heydari‐Majd et al., 2019; Jouki et al., 2014). The differences in the amounts of volatile nitrogen bases for different fish, treatments, and specific processing conditions have been reported as the limitations of these films (Özyurt et al., 2009). In fish and fishery products, in general, the total nitrogen volatility levels of freshly caught fish can be between 5 and 20 mg per 100 g of meat. However, the amount of 30–35 mg per 100 g of fish meat is acceptable for human consumption. Accordingly, the control treatment was not acceptable for consumption and the number of volatile nitrogen bases was less than 30 mg per 100 g in all treatments containing ZCEO.

## 4 CONCLUSION

In the present research, the best film properties were observed in the combination of PLA film + NC + 1% ZCEO. Furthermore, the results related to the quality of fish fillets indicated that the composite film of PLA/NC with ZCEO decelerated the increasing trend of oxidative spoilage indices over time while yielding better results by increasing the concentration of ZCEO. In conclusion, considering the more desirable physical and mechanical properties of PLA/NC containing ZCEO 1%, it seems that it can be utilized as an active, safe, customer‐friendly, and cost‐effective packaging for fish products with no environmental repercussion. Further studies with other fish and other nanocomposite films with essential oils may provide promising results in this regard.

## AUTHOR CONTRIBUTION

Nastaran Shakour: Investigation (lead); Methodology (lead); Writing Original Draft (lead); writing Review Editing (equal). Zhaleh Khoshkhoo: Supervision (lead); writing Review Editing (lead). Afshin Akhondzadeh Basti: Formal Analysis (lead). Ali Khanjari: Funding Acquisition (lead). Peyman Mahasti Shotorbani: Investigation (equal), software (lead).

## Data Availability

Data openly available in a public repository that issues datasets with DOIs.
